# Delayed Tension Pneumocephalus Following Frontal Sinus Fracture

**DOI:** 10.5811/cpcem.2021.9.53603

**Published:** 2022-01-06

**Authors:** Eddie X. Ortiz-Galloza, Bianca Arechiga, Jagdipak Heer, Daniel Quesada

**Affiliations:** Kern Medical, Department of Emergency Medicine, Bakersfield, California

**Keywords:** tension pneumocephalus, altered mental status

## Abstract

**Case Presentation:**

We describe a delayed case of tension pneumocephalus in a newly altered patient 21 days status-post auto-vs-pedestrian accident. After her initial hospital course, the patient was discharged to an acute rehabilitation facility in stable condition with Glasgow Coma Scale 15. The patient returned to the emergency department for an acute change in mental status.

**Discussion:**

Tension pneumocephalus is a neurosurgical and otolaryngological emergency.

## CASE PRESENTATION

**Image 1:**
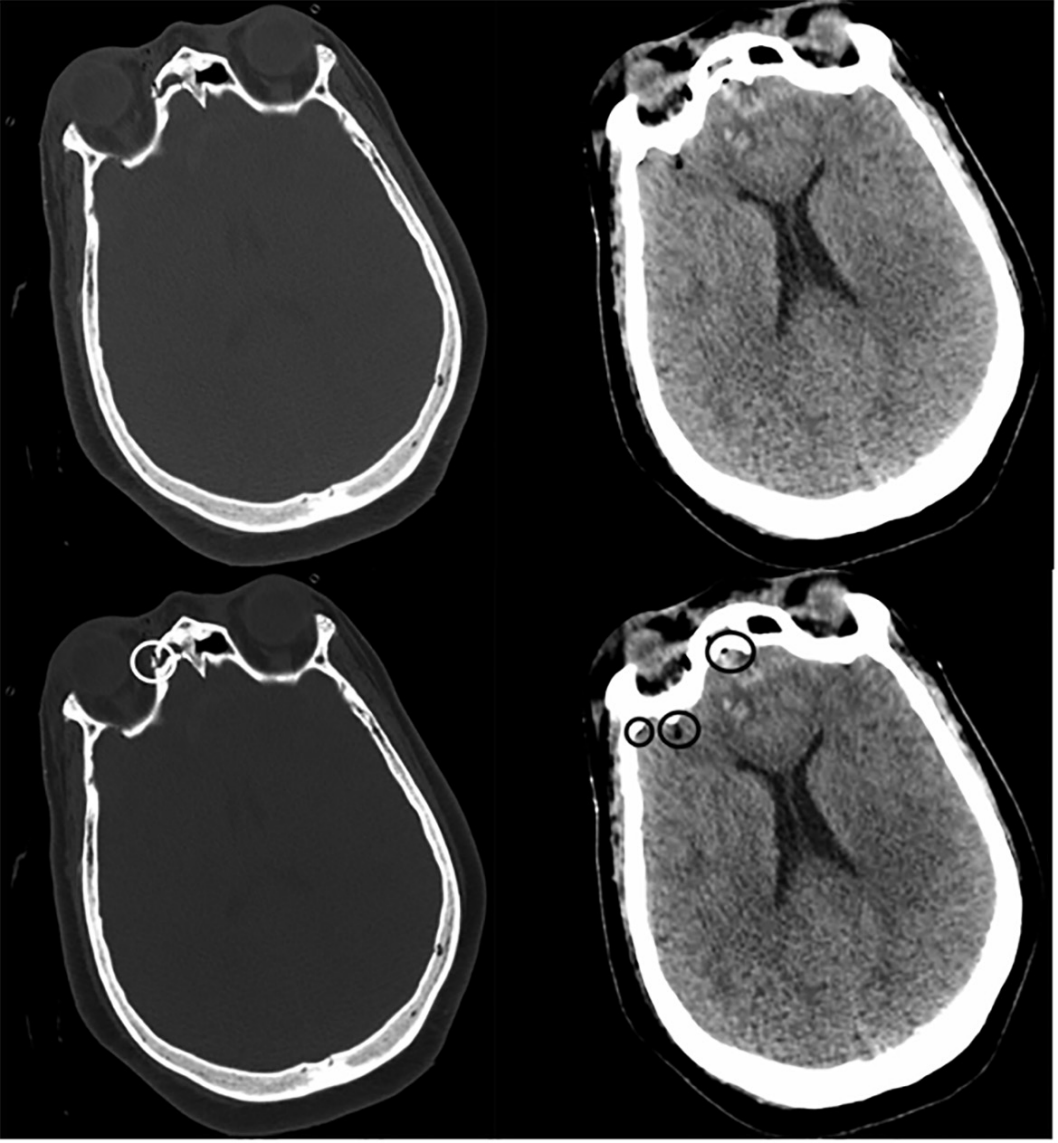
Computed tomography brain without contrast at initial presentation of a patient injured in an automotive-vs-pedestrian accident. LEFT: Bone window shows fractures in the medial wall of the right orbit (*white circle)*. RIGHT: Brain window shows small drops of pneumocephaly in the right frontal lobe (*black circles*).

A 72-year-old female with a history of auto-vs-pedestrian accident 21 days prior presented from an acute rehabilitation facility (ARF) for altered mental status. Nursing staff at the ARF reported that the patient had become gradually less responsive and interactive over a period of one hour prior to arrival. She was previously hospitalized for traumatic brain injury including right frontal lobe contusion, small right frontal lobe sub-arachnoid hemorrhage, small drops of pneumocephaly in the right frontal lobe, right anterior and posterior frontal sinus fractures extending to the medial aspect of the orbital roof, and a fracture of the medial wall of the right orbit (Image 1).

Vital signs included a heart rate 114 beats per minute, blood pressure 137/58 millimeters of mercury, respiratory rate 18 breaths per minute, and oxygen saturation 100% on room air. Physical exam revealed somnolence but arousing to minor stimulation, severe aphasia, Glasgow Coma Score (GCS) 11 (eyes 4, verbal 1, motor 6), right gaze deviation with inability to track left past the midline, and left hemiparesis of both extremities.

**Image 2:**
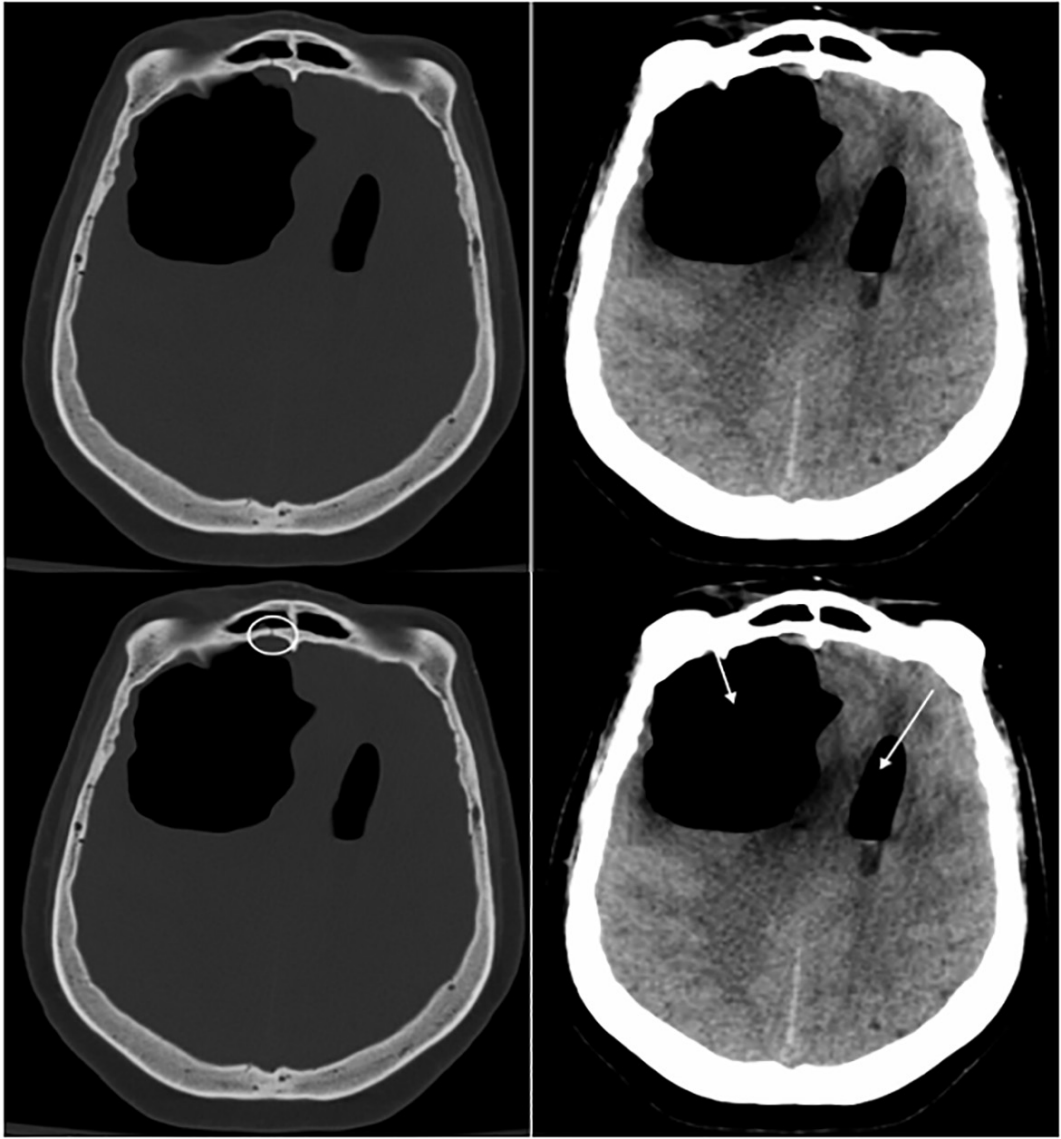
Computed tomography brain without contrast at presentation 21 days later. LEFT: Bone window shows a right frontal sinus posterior wall fracture (*white circle*). RIGHT: Brain window shows right frontal loculated pneumocephalus (*white arrows*) with extension into ventricular system and left midline shift.

Computed tomography (CT) of the brain showed interval development of right frontal loculated pneumocephalus measuring 6.6 × 5.5 × 4.9 centimeters exerting mass effect resulting in diffuse cerebral sulci effacement, 14 millimeters leftward subfalcine herniation, and suspected early right uncal herniation (Image 2). Neurosurgery and otolaryngology were consulted, and the patient subsequently went to the operating room for frontal bone repair, ethmoidectomy and closure of cerebrospinal-fluid leak. She had an uneventful recovery and was discharged to a skilled nursing facility with a GCS of 15, consistent with her mental status at the time of her initial discharge.

## DISCUSSION

This case demonstrates the complexity of a geriatric patient presenting with acute altered mental status. A broad differential diagnosis was considered. Infection and metabolic derangements were investigated; however, given the patient’s neurological exam and recent history of trauma, CT of the head was ordered. This case elucidates the potential complications that can occur when facial fractures are present and observation rather than surgical repair is chosen. Tension pneumocephalus is a neurosurgical and otolaryngological emergency. Treatment is surgical decompression and fracture repair by the respective specialties.^1^ The incidence of tension pneumocephalus associated with head trauma is less than 1%. However, the incidence increases to 8% with paranasal sinus or skull base fractures.^2^

**Educational Merit Capsule**

What do we already know about this clinical entity?

*Tension pneumocephalus (TP) is a surgical emergency. Paranasal sinus and skull base fractures have a significantly increased risk of developing TP*.

What is the major impact of the image(s)?

*These images reveal the potential complications that can occur when facial fractures are present and observation, rather than surgical repair, is chosen*.

How might this improve emergency medicine practice?

*Clinicians must repeat imaging on patients with a history of recent head trauma, specifically facial fractures, as tension pneumocephalus is a rare but possible diagnosis*.
